# Understanding the direct and indirect costs of patients with schizophrenia

**DOI:** 10.12688/f1000research.6699.2

**Published:** 2015-08-06

**Authors:** Kazuhiro Tajima-Pozo, María Jesús de Castro Oller, Adrian Lewczuk, Francisco Montañes-Rada

**Affiliations:** 1 Universidad Rey Juan Carlos de Madrid, Madrid, 28933, Spain; 2 Medical University of Warsaw, Warsaw, 61, Poland

**Keywords:** Schizophrenia costs, Europe, indirect and direct costs, disability, antipsychotic

## Abstract

***Background***: Schizophrenia is a disabling mental disorder with high prevalence and that usually  requires long-term follow-up and expensive lifelong treatment. The cost of schizophrenia treatment consumes a significant amount of the health services' budget in western countries.

***Objective***: The aim of the study was to find out about the costs related to schizophrenia across different european countries and compare them.

***Results***: Schizophrenia treatment costs an estimated 18 billion euros annually worldwide. The direct costs associated with medical help are only part of the total expenditure. The indirect costs are an equally (or even more)important part of the total cost. These expenses are related to the lack of productivity of schizophrenic patients and the cost that relatives have to bear as a result of taking care of their affected relatives.

***Conclusions***: Although data on the cost of schizophrenia may vary slightly between different european countries, the general conclusion that can be drawn is that schizophrenia is a very costly disorder. Not only because of direct costs related to medical procedures, but also due to the non-medical (indirect) costs. Together this suggests the need to investigate cost-efficient strategies that could provide a better outcome for schizophrenic patients, as well as the people who care for them.

## Introduction

### Why is schizophrenia such a costly disease?

Schizophrenia is a chronic psychiatric condition that affects around 1% of the population worldwide ^1– 4^. It is one of the most stigmatizing diseases of all time ^3, 5^. Schizophrenic disorders present with a wide range of symptoms, both positive and negative, leading to cognitive, social and functional impairment. Therefore most individuals with schizophrenia are considered disabled and claim benefits ^6^. The debilitating nature of the disorder means that patients receive lifelong treatment, and a large proportion of them need to be admitted to a hospital inpatient unit on multiple occasions throughout their lifetime ^1– 3^.

All of the above factors lead to the high costs associated not only with the treatment of schizophrenia but also related to social impairment ^[Bibr ref-3], 7^. By these we mean the inability to work and also the way that schizophrenia affects the patients´ environment. The need to take care of and support schizophrenic relatives is a major reason for members of their families to take sick leave or even sacrifice their own career ^3, 8^. All of this, plus the high expense associated with the newest antipsychotic drugs, makes the costs of schizophrenia management as high as 3% of the total healthcare budget of western countries ^1^.

The aim of this paper is to give a global view on the problem and to emphasize certain cost-inducing aspects of schizophrenia management by reviewing past research on the costs of schizophrenia management.

### Understanding the direct and indirect costs of schizophrenia – the clue to the problem

Regardless of the authors’ origin, they all agree unanimously that the costs associated with the treatment and care of patients with schizophrenia can be divided into two important groups: direct and indirect costs ^2– 4, 8, 9^. The direct costs of treating schizophrenia include cost of hospitalization (short- and long-term), outpatient follow-up, residential and day care, pharmaceutical interventions, laboratory testing and social security payments, whereas the indirect costs are mainly related to the loss of productivity ^1, 3, 10, 11^. The age of onset of the disorder, usually in the late teens or early 20s, can preclude patients from even starting to work ^12^. Later on, most of the patients receive benefits for incapacity for work due to disability ^6^. Nowadays, most schizophrenic patients receive a disability certificate and eventually do not work. Up to 80% of schizophrenic patients in the UK do not have paid employment ^3^. In Italy and Spain, three out of four patients with schizophrenia are excluded from the job market ^9, 13^. Thus, some authors consider the loss of productivity as accounting for the majority of the indirect costs ^4^.

Speaking of indirect costs, it is also important to consider the indirect cost associated with caregivers to schizophrenic patients, who contribute with their time and in-kind services ( Table 1). Therein lies the issue. The real number of people affected by schizophrenia is much bigger than just the number of the patients. According to some authors, direct and indirect costs are approximately equal ^14^, whereas others suggest that indirect costs can outnumber the direct ones up to three or four times ^11, 15^.

**Table 1.** Types of schizophrenia-related costs. Costs are divided in 3 groups: direct cost, indirect cost and intangible cost.

Type of costs	Direct	Indirect	Intangible
**Examples**	- hospitalisation (short-and long-term) - outpatient follow-up - residential and day care - drugs - laboratory testing - social security payments	- loss of productivity - the cost that the care-givers bear by contributing their time and in-kind services	- of non-financial nature - side-effects of the drugs - stress and anxiety caused by the disorder itself and the treatment process

Some authors also distinguish a third group of costs, called “intangible costs” ^3^. These are expenses of a non-financial nature. They try to accomplish the hard task of reflecting the patients´ quality of life, including side-effects of pharmaceutical interventions and stress and anxiety, both caused by the disease itself and also the treatment process. Although this group might have no direct financial impact, it is worth considering these factors as they probably affect the cooperation between patient and health providers ^16, 17^. Furthermore, we can also take into account the intangible costs of the caregivers of schizophrenic patients ^3^. Daily care of a schizophrenic relative can be a very challenging and exhausting experience ^3, 18^. Social stigma and the lack of sympathy and understanding may lead to anxiety and depression in caregivers as well as sufferers ^5^. This could damage the relationship between the caregiver and the caretaker, which may lead to an increased rate of patient deterioration and worse prognosis in the long-term ^18^.

### Data collection in relation to the aims of this article

The main part of data used in this article come from psychiatric wards in Spain, France, Sweden, Poland, United Kingdom and Ukraine ( Table 2 and Table 3). Data from recent USA research have also been included for comparison purposes. Although some of the methods used for data collection vary, depending on the country and researchers, the general idea of this article is to get a global view on the subject. Thus, some estimations can be made and their legitimacy is consistent as shown by the similarities between the results.

**Table 2.** Cost of schizophrenia in France, Spain, United Kingdom and USA are shown in Table 2. Total cost is defined as a sum of the direct and indirect cost. Direct, indirect and total costs are defined by authors across different articles with similar criteria which have been discussed in the Introduction section. Total cost is defined as a sum of the direct and indirect cost. The table also contains the proportion of the cost of pharmaceutical treatment (Drug cost) in relation to the total cost.

Country	Direct costs	Indirect costs	Total cost	Drug cost in relation to the total cost	Authors and year
**France**	1 581 milion **€**	2 214 milion **€**	3 534 milion **€**	16,1%	Emmanuelle Sarlon *et al.* 2012 (data comes from 1998–2002)
**Spain**	1 044 milion **€**	926 milion **€**	1 970 milion **€**	12,8%	Juan Oliva-Moreno *et al.* 2006 (data comes from 2002)
**UK**	714 milion £	1886 milion £	2 600 milion £	4%	Martin Knapp, 1997 (data comes from 1992–1993)
**USA**	32 051 milion $	32 378 milion $	64 429 milion $	8%	Joseph P. McEvoy 2007 (data by Wu *et al.* 2002)

**Table 3.** Examples of schizophrenia costs in Poland (in the city of Poznan) and Ukraine (in the city of Lviv) – comparison. The data coming from these two eastern European countries ***only includes direct costs*** (total cost=total direct cost) 50 patients were included in the search in Poland and 58 patients in Ukraine. (Tomasz Zaprutko *et al.*, 2014; data comes from years 2010–2011) ^4^.

City	Number of patients	Total direct cost	Pharmacotherapy cost in relation to the total cost
**Poznan (Poland)**	50	160,572.08 **€**	6,60%
**Lviv (Ukraine)**	58	30,943.37 **€**	6,43%

## Methods

The research data for this article was collected by the use of the PubMed database in April 2015, having used key words: “ *schizophrenia costs in Europe”, “(indirect and direct costs) schizophrenia”, “schizophrenia costs worldwide”, “schizophrenia costs United States”, “schizophrenia and disability”, “antipsychotic treatment in Europe”* as a part of the abstract, title or included anywhere in the whole paper. We analyzed 41 articles that we managed to find according to the criteria we adopted.

We have taken into account comparable data, e.g. annual expenses on schizophrenia treatment, expenses per capita on schizophrenia treatment. In addition, we have taken into account these possible differences concerning data collecting methods used by the particular researchers, as well as different years in which the researchers conducted their studies, the comparison can only estimate the true cost of schizophrenia treatment.

We wanted to include only the latest data coming from research conducted after year 2000. Due to the fact that there has been very little research done in this field in general we decided to analyze papers from the whole PubMed dataset. We did however exclude the earliest data (Australia 1976; USA 1975, 1985; Netherlands 1989) since we found them irrelevant (for example, at that time second-generation antipsychotic drugs were not used and they account for a significant part of medication costs).

The nature of data varies between different authors. Some articles give total amounts of money in relation to the direct and indirect schizophrenia treatment costs in a particular country. The others give numbers per patient. The percentage approximations of pharmacological costs are also present, directly obtained from papers.

We included only those articles showing general information about costs of schizophrenia across different countries, and excluded those ones related only to specific services, like acute inpatient units, where stated costs did not include rehabilitation expenses or full treatment options, what could lead to important bias when comparing pharmacological expenses or indirect costs.

## Results and discussion

This paper points at the magnitude of the problem of schizophrenia treatment costs and estimates the huge impact that this mental disorder has on patients´ environment and society at a financial level across several different countries ^3, 7, 16^.

What definitely strikes attention when it comes to available data is that the estimated indirect cost represents a significant part of the total cost of schizophrenia. It is particularly important to bear that in mind in order to manage schizophrenia efficiently.

The other conclusion that can be drawn from our research is that the cost of pharmaceutical treatment doesn’t contribute significantly to the total cost of treatment ^7, 16^. This statement is equally consistent for both Western ( Table 2 shows percentages which range from 4% in UK to 16,1% in France; and Eastern European countries such as Poland and Ukraine ( Table 3). These results may suggest important differences on the cost of non-pharmaceutical care provided across countries ^1^, which need further investigation.

### Limitations of study and recommendations for future research

Collected data across different studies vary in terms of the number of patients included or hospitals involved across different investigations. An exact comparison between all papers is obviously not possible, but certain estimations can be made.

In some countries, like France and UK, the indirect costs outnumber the direct ones ( Table 2) ^4, 15^. On the other hand, results coming from Spain and USA suggest that both types of cost are equally important in total ^12, 14^. To better understand this result we need to consider the differences across particular studies regarding design and methods used. For instance, the approach to calculate the cost of lost workforce varies between the countries, which could lead to some of the differences observed. In Spain, the official registries do not reflect the work force lost by people who have never even started a job – and this group of people accounts for a large proportion of schizophrenic patients since the onset of the disorder (and so the problems with getting or maintaining employment) may occur early in life, before young people begin their career ^14^.

Therefore, it could be possible that the indirect costs are even higher in the countries who register its workforce in this way.

### How to improve the situation?

Many authors raise the issue of patients´ adherence to prescribed therapy. An optimal control of schizophrenic symptoms is proven to lead to fewer numbers of hospitalizations and less need to use other approaches of formally organized patient care ^19^. Although good adherence to treatment means no reduction in the budget for pharmaceutical interventions, this cost does not seem to represent a significant percentage when compared to total cost of the illness (drugs costs vs. total costs – see Table 2, Table 3). Good psychoeducational programs and building insight about the disease and its management makes patients feel more secure and thus more cooperative ^1, 3^.

When the symptoms of schizophrenia remain under control, patients experience a smaller risk of mental impairment and social exclusion which extends to participation in work opportunities ^20^. So it is of crucial importance to investigate whether better control of symptoms could allow patients to get a chance to attain and keep a job, and how this fact could affect their quality of life. Moreover, we should consider how this could be used to lighten the burden of care that relatives and caregivers experience and thus reduce the indirect costs which make up a significant part of the costs of this illness.

Therefore, it is our suggestion that the future of schizophrenia treatment should address more carefully important elements of the financial aspects of the disease, such as cost-efficacy of treatment, including psychological therapies and psychoeducational approaches for both patients and their families. A wider view on the matter is needed.
