# Supplementary material for: Understanding the direct and indirect costs of patients with schizophrenia
Source: F1000Res. 2015 Aug 6;4:182. Originally published 2015 Jul 6. [Version 2] doi: 10.12688/f1000research.6699.2 (PMC4544407; doi:10.12688/f1000research.6699.2)
Supplement: Peer review history

# Peer review: Referee response for version 2

**DOI:** 10.5256/f1000research.7376.r9897

**Published:** 2015-08-07

**Authors:** Lucas Giner

**Affiliations:** 1 Department of Psychiatry, Faculty of Medicine, University of Seville, Seville, Spain

I think the article may have those tables proposed by the authors.

I have read this submission. I believe that I have an appropriate level of expertise to confirm that it is of an acceptable scientific standard.

# Peer review: Referee response for version 2

**DOI:** 10.5256/f1000research.7376.r9910

**Published:** 2015-08-06

**Authors:** Mayumi Okuda

**Affiliations:** 1 Department of Psychiatry, New York State Psychiatric Institute, The College of Physicians and Surgeons, Columbia University Medical Center, New York, NY, USA

A few concerns that I have:

1. Was not fully addressed, some phrases still contain the term "schizophrenic." The text should be more thoroughly reviewed.
2. Was not addressed. It's a bit confusing whether the paper aims to describe findings from economic studies in Europe vs worldwide. Not clearly stated where the 18 billion figure comes from. Not sure then why the study by Frey 2014 in Germany was not included.

I have read this submission. I believe that I have an appropriate level of expertise to confirm that it is of an acceptable scientific standard.

# Peer review: Referee response for version 1

**DOI:** 10.5256/f1000research.7196.r9352

**Published:** 2015-08-03

**Authors:** Mayumi Okuda

**Affiliations:** 1 Department of Psychiatry, New York State Psychiatric Institute, The College of Physicians and Surgeons, Columbia University Medical Center, New York, NY, USA

The article offers a concise, brief summary of an important topic. The following are minor revisions that will improve the manuscript

1. Changing the terminology "schizophrenic patients" to "patients with schizophrenia" or "individuals with schizophrenia".
2. In the abstract the objective states the aim is to report on the costs of schizophrenia across different European countries, yet the results states "18 billion euros worldwide." Given that the manuscript reviews economic studies in Europe, it should be modified to focus on European findings.
3. The last few sentences in paragraph 3 of the introduction describe limitations of this review; these sentences should be described in the methods section. It is not completely clear to me what the authors are referring to when they mention "other data" that could not be compared.
4. A paragraph following the introduction or within the introduction detailing the types of costs that are typically measured in these type of studies could replace table 1.
5. Detailing the types of costs that were measured in the different studies would be a valuable contribution to Table 2.

I have read this submission. I believe that I have an appropriate level of expertise to confirm that it is of an acceptable scientific standard.

# Response

**Published:** 2015-08-03

**Authors:** Kazuhiro Tajima-Pozo

**Affiliations:** Hospital Fundacion Alcorcon, Spain

Thanks for your review, we have made the following changes in our latest version

1. Change the terminology "schizophrenic patients" to "patients with schizophrenia" or "individuals with schizophrenia".
2. The last few sentences in paragraph 3 have been moved to the methods section.
3. A brief paragraph following the introduction of table 1 have been included.
4.  A brief paragraph following the introduction of table 2 have been included.

Best regards and thanks for your quick and good review of our paper

# Peer review: Referee response for version 1

**DOI:** 10.5256/f1000research.7196.r9406

**Published:** 2015-07-16

**Authors:** Lucas Giner, Diego De La Vega

**Affiliations:** 1 Department of Psychiatry, Faculty of Medicine, University of Seville, Seville, Spain; 2 Unidad de Salud Mental Comunitaria de Huelva, Complejo Hospitalario Universitario de Huelva, Huelva, Spain

Brief and nice review of an important topic related to economic cost of schizophrenia. Results are presented clear. However, I think that tables with examples are not necessary and should be considered to be deleted. The text does not need any change in my consideration.

We have read this submission. We believe that we have an appropriate level of expertise to confirm that it is of an acceptable scientific standard, however we have significant reservations, as outlined above.

# Response

**Published:** 2015-07-16

**Authors:** Kazuhiro Tajima-Pozo

**Affiliations:** Hospital Fundacion Alcorcon, Spain

Thanks for your quick review and your comments.

# Response

**Published:** 2015-07-20

**Authors:** Kazuhiro Tajima-Pozo

**Affiliations:** Hospital Fundacion Alcorcon, Spain

According to the reviewer comments, we have deleted table 4, and we have maintained table 1, 2 and 3. I hope that this changes could contribute to the final acceptance of the paper.
